# NPM-ALK Is a Key Regulator of the Oncoprotein FOXM1 in ALK-Positive Anaplastic Large Cell Lymphoma

**DOI:** 10.3390/cancers11081119

**Published:** 2019-08-06

**Authors:** Moinul Haque, Jing Li, Yung-Hsing Huang, Meaad Almowaled, Carter J. Barger, Adam R. Karpf, Peng Wang, Will Chen, Suzanne D. Turner, Raymond Lai

**Affiliations:** 1 Department of Laboratory Medicine and Pathology, University of Alberta, Edmonton, AB T6G2R3, Canada; 2 Electron Microscopy Center, Basic Medical Science College, Harbin Medical University, Harbin 150080, Heilongjiang, China; 3 Eppley Institute and Fred & Pamela Buffett Cancer Center, University of Nebraska Medical Center, Omaha, NE 68198, USA; 4 Department of Hematology, University of Alberta, Edmonton, AB T6G2R3, Canada; 5 Division of Cellular and Molecular Pathology, Department of Pathology, University of Cambridge, Cambridge CB20QQ, UK; 6 Department of Oncology, University of Alberta, Edmonton, AB T6G2R3, Canada

**Keywords:** anaplastic large cell lymphoma, NPM-ALK, FOXM1, gene regulation, thiostrepton

## Abstract

Forkhead Box M1 (FOXM1) is an oncogenic transcription factor implicated in the pathogenesis of solid and hematologic cancers. In this study, we examined the significance of FOXM1 in NPM-ALK-positive anaplastic large cell lymphoma (NPM-ALK + ALCL), with a focus on how it interacts with NPM-ALK, which is a key oncogenic driver in these tumors. FOXM1 was expressed in NPM-ALK + ALCL cell lines (5/5), patient samples (21/21), and tumors arising in NPM-ALK transgenic mice (4/4). FOXM1 was localized in the nuclei and confirmed to be transcriptionally active. Inhibition of FOXM1 in two NPM-ALK + ALCL cells using shRNA and pharmalogic agent (thiostrepton) resulted in reductions in cell growth and soft-agar colony formation, which were associated with apoptosis and cell-cycle arrest. FOXM1 is functionally linked to NPM-ALK, as FOXM1 enhanced phosphorylation of the NPM-ALK/STAT3 axis. Conversely, DNA binding and transcriptional activity of FOXM1 was dependent on the expression of NPM-ALK. Further studies showed that this dependency hinges on the binding of FOXM1 to NPM1 that heterodimerizes with NPM-ALK, and the phosphorylation status of NPM-ALK. In conclusion, we identified FOXM1 as an important oncogenic protein in NPM-ALK+ ALCL. Our results exemplified that NPM-ALK exerts oncogenic effects in the nuclei and illustrated a novel role of NPM1 in NPM-ALK pathobiology.

## 1. Introduction

Forkhead box M1 (FOXM1) is a member of the Forkhead (FOX) family of transcription factors, which all share a highly conserved winged-helix DNA-binding domain [1]. FOXM1 was originally identified as an important factor driving the proliferation of lymphocytes [2]. Specifically, FOXM1 was found to facilitate progression to the G2/M phase by upregulating cell-cycle regulators such as Cyclin B1 (*CCNB1*), S-Phase Kinase Associated Protein 2 (*SKP2*), and Aurora Kinase A (*AURKA*) [3]. The oncogenic potential of FOXM1 has been demonstrated in various solid tumor models [1]. FOXM1 has been shown to exert its oncogenic effects via several mechanisms, including its promotion of cell cycle progression, tissue invasion, angiogenesis, metastasis, withdrawal from cellular senescence, resistance to DNA damaging agents, and stem cell self-renewal [1,4]. The expression and biological significance of FOXM1 in hematologic malignancies have not been extensively investigated. Few published studies have specifically addressed the functional importance of FOXM1 in hematologic cancers. These include precursor B-cell acute lymphoblastic leukemia [5], T-cell acute lymphoblastic leukemia [6,7], plasma cell myeloma [8], diffuse large B cell lymphoma [9], and acute myeloid leukemia [10]. In these studies, experimental results from FOXM1 knockout mice and in vitro studies in which FOXM1 was inhibited by a using pharmacologic agent (i.e., thiostrepton) or gene knockdown support the concept that FOXM1 contributes to the oncogenicity of these cancers. Nonetheless, the mechanism by which FOXM1 mediates its oncogenic effects in these cancers is not well-understood.

NPM-ALK-positive anaplastic large cell lymphoma (NPM-ALK+ ALCL) is a distinct subtype of non-Hodgkin lymphoma originating from lymphoid cells of T/null cell immunophenotype [11]. NPM-ALK expression is the consequence of a t(2;5)(p23;q35) chromosomal translocation, results in the fusion of the N-terminus of nucleophosmin 1 (NPM1) to the C-terminus of anaplastic lymphoma kinase (ALK), and the generation of the NPM-ALK fusion protein that is a constitutively active tyrosine kinase [11]. Mounting evidence has suggested that NPM-ALK is the key oncogenic driver of NPM-ALK + ALCL. NPM-ALK binds to and activates a host of cellular signaling pathways including those of the JAK/STAT [12], PI3K/AKT [13], and MAPK [14]. More recent studies have suggested that NPM-ALK can exert oncogenic effects via a host of additional mechanisms, including its ability to (1) upregulate cytokines that can promote tumor growth and metastasis, (2) enhance the anti-tumor immune response, adaption to hypoxia, angiogenesis, and (3) deregulate DNA methylation, DNA mismatch repair, metabolism, and inhibit various tumor suppressors (e.g., SHP1 and STAT1) [15]. Additional studies also suggest that NPM-ALK can promote cancer stemness by upregulating the expression of the embryonic stem cell marker SOX2 [16]. Thus, the multi-faceted nature of NPM-ALK in driving oncogenesis has gradually emerged.

The expression and function of FOXM1 have never been investigated in NPM-ALK + ALCL. In this paper, we report for the first time that FOXM1 is highly expressed in NPM-ALK + ALCL and contributes to its oncogenesis. Importantly, our studies reveal a novel functional interaction involving FOXM1 and NPM-ALK mediated by NPM1. Furthermore, our study shows that NPM-ALK increases the transcriptional activity of FOXM1. Our findings, thus, reveal another important facet of how NPM-ALK promotes oncogenesis.

## 2. Results

### 2.1. NPM-ALK + ALCL Cells Express Elevated FOXM1

**Figure 1:**
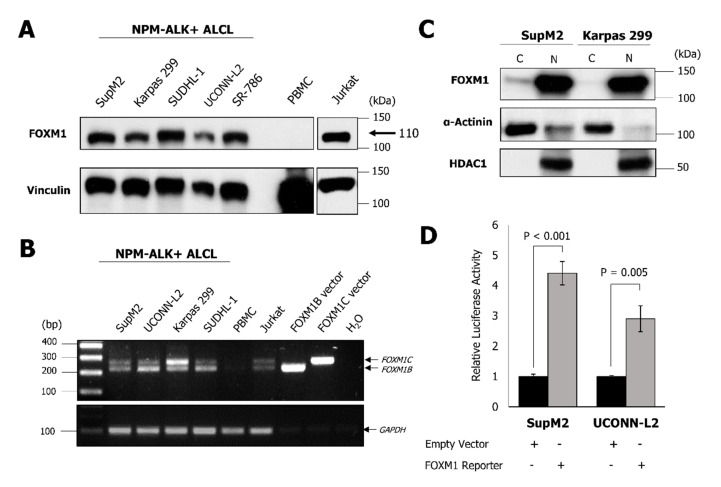
FOXM1 is expressed in NPM-ALK+ ALCL cell lines. (**A**) Western blot analysis for FOXM1 expression in the indicated cell lines. (**B**) RT-PCR for transcript levels of FOXM1B and FOXM1C mRNA in the indicated cell lines. (**C**) Western blot analysis of cytoplasmic (C)/nuclear (N) fractions of two NPM-ALK+ ALCL cell lines. (**D**) Luciferase reporter assay with a FOXM1 reporter was used to assess FOXM1 transcriptional activity in the indicated cell lines. Data are representative of at least three biological replicates. PBMC; peripheral blood mononuclear cells.

NPM-ALK + ALCL cell lines were assessed for expression of FOXM1 by using a Western blot. As shown in Figure 1A, FOXM1, detected at approximately 110 kDa, which is concordant with its previously reported molecular weight, was highly expressed in all five cell lines examined [17]. In contrast, cell lysates harvested from peripheral blood mononuclear cells (PBMC) from a healthy individual showed no detectable FOXM1. Jurkat (a T-cell acute lymphoblastic leukemia cell line), which has previously been reported to express a high level of FOXM1 [18], was found to express FOXM1 at a level comparable to that of NPM-ALK + ALCL cell lines.

In order to determine whether both oncogenic FOXM1 isoforms (i.e., FOXM1B and FOXM1C) are expressed in NPM-ALK + ALCL, an RT-PCR was performed using a specific primer set that is designed to detect both FOXM1B and FOXM1C [19] (since these isoforms are known to share a similar molecular weight and cannot be readily distinguished from each other by Western blot). Expression of both isoforms was detectable in all four NPM-ALK+ ALCL cell lines illustrated but not PBMC (Figure 1B). Detection of both FOXM1 isoforms in SR-786 was also identified (data not shown). Jurkat cells and the PCR products from commercially available FOXM1B and FOXM1C expression vectors served as positive controls.

Since FOXM1 is a transcription factor, we questioned if this protein is transcriptionally active in NPM-ALK + ALCL. In support of the concept that FOXM1 is transcriptionally active, FOXM1 was found to be localized to the nuclei of NPM-ALK + ALCL cells, as shown by nuclear/cytoplasmic fractionation experiments (Figure 1C). Histone deacetylase (HDAC1) and α-Actinin served as the controls for the efficiency of the nuclear/cytoplasmic fractionation protocol. Furthermore, following transfection of a FOXM1 luciferase reporter, containing FOXM1 consensus sequences, into NPM-ALK + ALCL cells, a significantly higher level (*p* < 0.005) of luciferase activity was observed compared to the negative control (Figure 1D).

**Figure 2:**
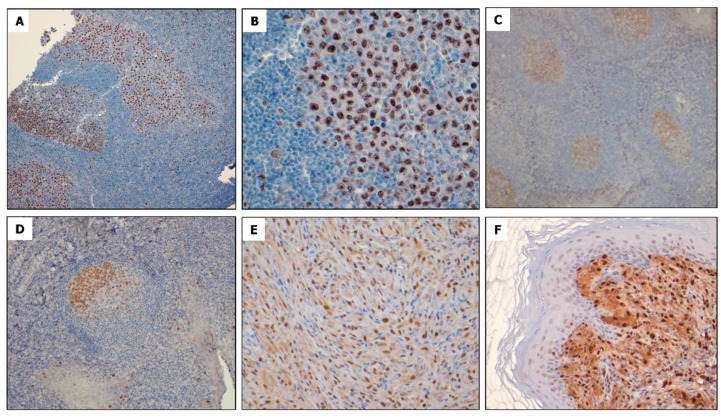
FOXM1 is expressed in primary tumors of patients diagnosed with ALK + ALCL and tumors developing in transgenic mice expressing NPM-ALK in lymphocytes. (**A**) Immunohistochemical staining of a representative case of ALK + ALCL shows FOXM1 expression in reactive germinal center lymphocytes and large lymphoma cells infiltrating the sinus of a lymph node (immunoperoxidase, 100×). (**B**) High magnification (400×) of the same case of ALK + ALCL highlighting strong FOXM1 staining in the nuclei of lymphoma cells. In comparison, the surrounding benign, small lymphocytes are negative for FOXM1. (**C**) Immunohistochemical studies of a reactive tonsil show that FOXM1 expression is largely restricted to germinal centers. (**D**) High magnification of the same case of tonsil revealed that FOXM1 staining was largely restricted to centroblasts in germinal centers (400×). (**E**) and (**F**) ALK + ALCL tumors arising from NPM-ALK transgenic mice express nuclear and cytoplasmic FOXM1 in the neoplastic cell population, even though FOXM1 staining is stronger in the nucleus when compared to the cytoplasm (400×).

To evaluate FOXM1 expression in primary NPM-ALK + ALCL tumors, immunohistochemistry (IHC) was conducted in 21 cases of formalin-fixed, paraffin-embedded tissue. In all cases examined, high expression of nuclear FOXM1 staining was identified in a subset of neoplastic lymphoid cells, whereas surrounding non-malignant small lymphocytes showed no definitive staining (Figure 2A,B). In a reactive tonsil, FOXM1 immunostaining was located mostly in centroblasts in the germinal centers and rare small lymphocytes in the mantle zones, but the inter-follicular spaces were largely negative (Figure 2C,D). Similar results were obtained on IHC of four tumors arising in NPM-ALK transgenic mice, which is from a study model previously published [20]. FOXM1 was found to be highly expressed in the nuclei of these ALK-expressing tumor cells with the surrounding benign cells being negative (Figure 2E,F).

### 2.2. FOXM1 Downregulation Inhibits Cell Growth and the Clonogenicity of NPM-ALK + ALCL Cell Lines

**Figure 3:**
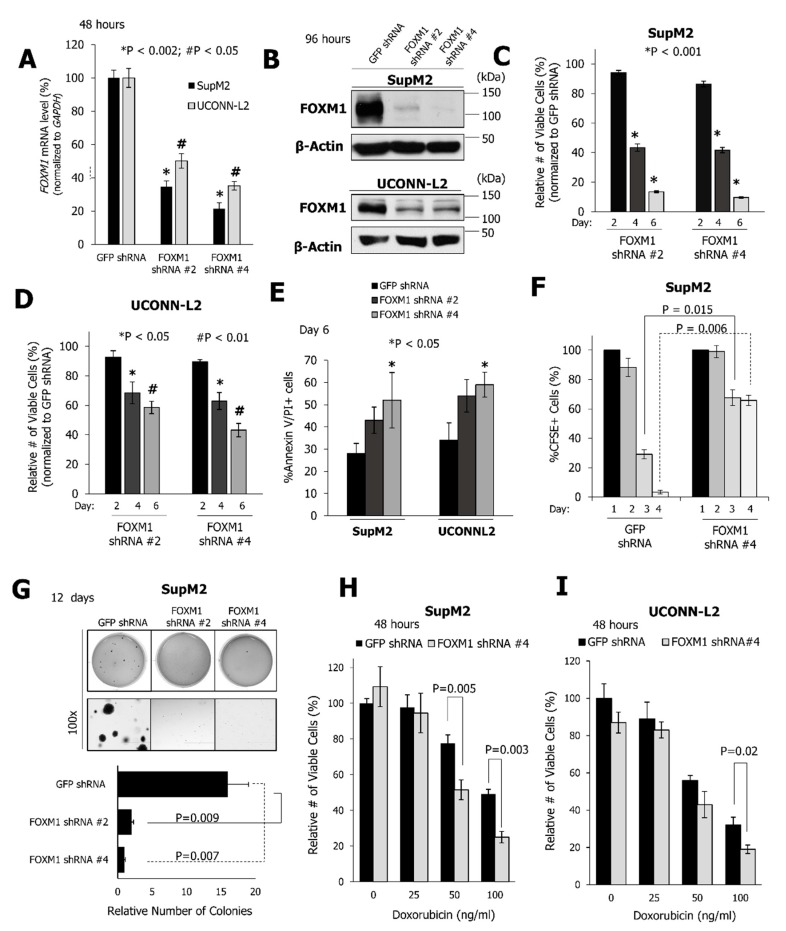
FOXM1 promotes cell growth and tumorigenicity of NPM-ALK + ALCL. (**A**) The FOXM1 transcript levels in SupM2 and UCONN-L2 cells 48 h following lentiviral transduction with one of two FOXM1-specific or a GFP-specific shRNA. (**B**) FOXM1 expression levels detected by a Western blot in SupM2 and UCONN-L2 cells 96 h following shRNA transduction with the indicated shRNA-containing vectors. β-actin was used as the loading control. (**C**,**D**) The relative number of viable cells (normalized to GFP shRNA) following trypan blue exclusion of SupM2 and UCONNL-2 cells 2, 4, and 6 days following transduction to express either GFP or FOXM1 shRNA. Results are normalized to the values of the GFP shRNA. (**E**) Annexin V/PI staining. (**F**) Proportion of cells with CFSE over the course of four days following GFP-specific or FOXM1-specific shRNA transduction. (**G**) Soft agar colony formation assay of NPM-ALK+ ALCL cells transduced to express either GFP-specific or FOXM1-specific shRNA. (**H**,**I**) MTS assay was used to measure the number of viable SupM2 cells (H) or UCONN-L2 cells (**I**) following transduction with the indicated shRNA in the presence of increasing doses of doxorubicin as indicated. Data are representative of three biological replicates for SupM2 cells and two biological replicates for UCONN-L2 cells.

To understand the biological significance of FOXM1 in NPM-ALK + ALCL, FOXM1 expression was suppressed in SupM2 and UCONN-L2 cells using two different short hairpin RNAs (shRNA). As shown in Figure 3A,B, efficient FOXM1 knockdown was achieved by using both shRNA species, even though the efficiency of knockdown was higher in SupM2 cells as compared to UCONN-L2 cells. shRNA-induced downregulation of FOXM1 resulted in the inhibition of the growth of SupM2 and UCONN-L2 cells on days four and six, as determined following trypan blue exclusion (Figure 3C,D). Cells infected with shRNA against the green fluorescence protein (GFP) served as the negative control. On day six, the decrease in the number of viable SupM2 cells ranged from 80% to 90%. shRNA mediated knockdown of FOXM1 in UCONN-L2 cells also resulted in a significant decrease in cell growth (~50% on day 6), even though the extent was not as profound as that seen in SupM2 cells, likely due to the fact that inhibition of FOXM1 in this cell line was not as efficient.

In addition to reducing the number of viable cells (normalized to GFP shRNA control), suppression of FOXM1 resulted in a significant increase in apoptosis of both SupM2 and UCONN-L2 cells, as defined by positive staining for both Annexin V and propidium iodide (PI) (*p* < 0.05, Figure 3E). These functional effects correlate with downregulation of Survivin, as well as increased cleavage of Caspase 3 and PARP (Figure S1). Since FOXM1 is known to be a facilitator of cell cycle progression, the impact of FOXM1 knockdown on cell proliferation was assessed using the CFSE assay, as described in Supplemental Materials and Methods. As shown in Figure 3F and Figure S2, the frequency of cell division, which is inversely proportional to the CFSE content, was decreased following suppression of FOXM1 expression in both cell lines. Furthermore, expression of the FOXM1 shRNA in either cell line resulted in a significant reduction in colony formation when compared to cells expressing GFP shRNA (Figure 3G and Figure S3). Moreover, FOXM1 knockdown sensitized cells to doxorubicin-induced cell growth inhibition on exposure to either 50 ng/mL or 100 ng/mL of doxorubicin for SupM2 cells, and 100ng/mL for UCONN-L2 cells (Figure 3H,I).

### 2.3. Pharmacological Inhibition of FOXM1 Inhibits the Growth and Clonogenicity of NPM-ALK + ALCL Cell Lines

**Figure 4:**
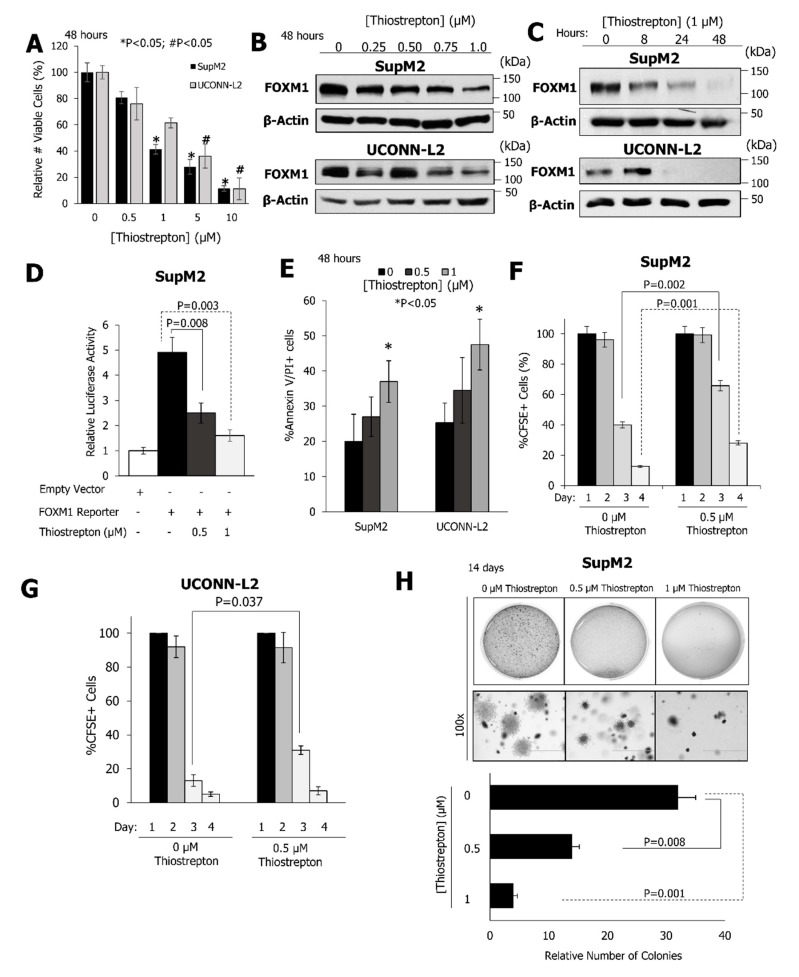
Pharmacological inhibition of FOXM1 inhibits cell growth and tumorigenicity. (**A**) The indicated cell lines were exposed for 48 h to thiostrepton, before the number of viable cells (normalized to DMSO) was assessed by an MTS assay. (**B**) Western blot of FOXM1 expression 48 h following exposure to thiostrepton in a dose-dependent manner. β-actin was used as a loading control. Anti-FOXM1 immunoblots were exposed for 1 min. (**C**) Western blot for expression levels of FOXM1 following exposure to 1 µM thiostrepton for the indicated times. Note that the exposure anti-FOXM1 immunoblots were exposed for 15–30 s. (**D**) Levels of luciferase activity in cells transiently transfected to express a FOXM1 promoter-luciferase reporter construct incubated in the presence or absence of thiostrepton for 48 h. (**E**) The fraction of cells to have undergone apoptosis, as defined by the co-expression of Annexin V and PI, in response to increasing doses of thiostrepton for 48 h. (**F**,**G**) Proportion of cells retaining CFSE following thiostrepton treatment for three or four days at the indicated dosages. (**H**) Colony formation in soft agar following incubation with increasing concentrations of thiostrepton for 48 h. The top panel shows overall colony growth in the plates with a 100x magnification while the lower panel shows colony counts. Data are representative of three biological replicates for SupM2 cells and two biological replicates for UCONN-L2 cells.

Thiostrepton, which is a pharmacological inhibitor of FOXM1 [21], potently inhibited the growth of SupM2 and UCONN-L2 cells with an IC50 of 0.89 µM and 1.22 µM, respectively, following 48 h of exposure to the compound (Figure 4A). Thiostrepton led to a potent and dose-dependent reduction in the expression of the FOXM1 protein in SupM2 and UCONN-L2 cell lines (Figure 4B) with complete inhibition of FOXM1 expression seen by 48 h (Figure 4C). To verify that the reduction in the viable cell number was due to thiostrepton through FOXM1 inhibition, the SupM2 cell line stably transfected with a FOXM1C tetracycline-inducible vector was treated with doxycycline for 24 h, which was followed by inhibition of FOXM1 with various doses of thiostrepton for 48 h. Cells with increased FOXM1 expression demonstrated significantly greater resistance to thiostrepton (Figure S4). Moreover, on transfection of a luciferase reporter construct containing FOXM1 consensus sequences, a significant and dose-dependent reduction in luciferase activity was observed upon treatment with thiostrepton (Figure 4D). Concomitantly, thiostrepton induced apoptosis as well as inhibition of cell division (i.e., CSFE staining) and soft agar colony formation of NPM-ALK + ALCL cell lines (Figure 4E–H).

### 2.4. FOXM1 Induces Phosphorylation of NPM-ALK and STAT3 in NPM-ALK + ALCL

**Figure 5:**
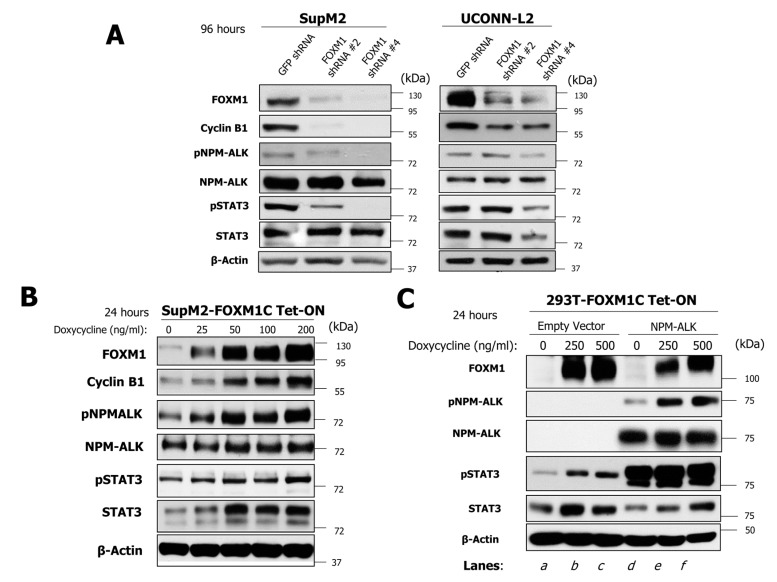
FOXM1 positively regulates the NPM-ALK/STAT3 signaling axis. (**A**) Western blot analysis of the indicated proteins in SupM2 and UCONN-L2 cells treated with two individual FOXM1 shRNA for 96 h. (**B**) Expression of the indicated proteins in SupM2 cells following exposure to increasing concentrations of doxycycline for 24 h, which activates expression of FOXM1. β-actin was used as a loading control. (**C**) Western blot analysis for expression of proteins in cells over-expressing FOXM1 in a dox-inducible manner in 293T cells transiently transfected to express NPM-ALK. Western blot data are representative of at least two biological replicates for each cell line.

We next investigated the mechanisms by which FOXM1 mediates its oncogenic effects in NPM-ALK + ALCL cells. Since the NPM-ALK/STAT3 signaling axis is the key oncogenic driving force in NPM-ALK + ALCL [11], we tested if the expression of FOXM1 carries any substantial impact on this signaling axis. As shown in Figure 5A, lentiviral shRNA knockdown of FOXM1 in SupM2 and UCONN-L2 cells resulted in a dramatic downregulation of Cyclin B1, which is a known downstream target of FOXM1 [22]. In the same experiment, downregulation of FOXM1 effectively inhibited the phosphorylation of NPM-ALK (i.e., pALK), without substantially modulating its total protein level. Similarly, phosphorylation of STAT3 at Y705 (i.e., pSTAT3) was also dramatically down regulated in both cell lines. Interestingly, the total STAT3 protein level was not substantially modulated in SupM2 whereas both pSTAT3 and total STAT3 decreased concurrently in the UCONN-L2 cells treated with FOXM1 shRNA #4. Cells treated with lentiviral shRNA against GFP served as the negative control.

To further substantiate that FOXM1 activates the NPM-ALK/STAT3 axis, we enforced the expression of a FOXM1 construct (i.e., FOXM1C) cloned in a tetracycline-inducible system in SupM2 cells. As shown in Figure 5B, the addition of increasing doses of doxycycline induced FOXM1 protein expression in a dose-dependent manner. Accordingly, Cyclin B1, which is a known downstream target FOXM1 [22], also increased in a dose-dependent manner. With this in vitro system, FOXM1 was found to upregulate pALK in a dose-dependent manner, while the total NPM-ALK level only slightly increased in this experiment. In the same experiment, we also found both pSTAT3 and total STAT3 to be substantially elevated.

To determine whether this FOXM1-mediated activity is specific to NPM-ALK + ALCL, 293T cells were stably transfected with a FOXM1C doxycycline-inducible vector in the presence or absence of NPM-ALK (Figure 5C). With increasing doses of doxycycline, a dose-dependent increase of FOXM1 expression was observed. In the presence of NPM-ALK (lanes d–f), a dose-dependent increase in phosphorylation of NPM-ALK could be found. Consistent with previous observations, STAT3 was phosphorylated in the presence of NPM-ALK even though the presence of FOXM1 did not increase this further. However, an appreciable elevation in the expression of total STAT3 was detected. Interestingly, in the absence of NPM-ALK (lanes a–c), upregulation of FOXM1 also resulted in appreciable increases in both pSTAT3 and STAT3, which suggests that FOXM1 can activate the STAT3 signaling pathway in an NPM-ALK-independent manner in 293T cells.

### 2.5. NPM-ALK Induces Transcriptional Activity of FOXM1

**Figure 6:**
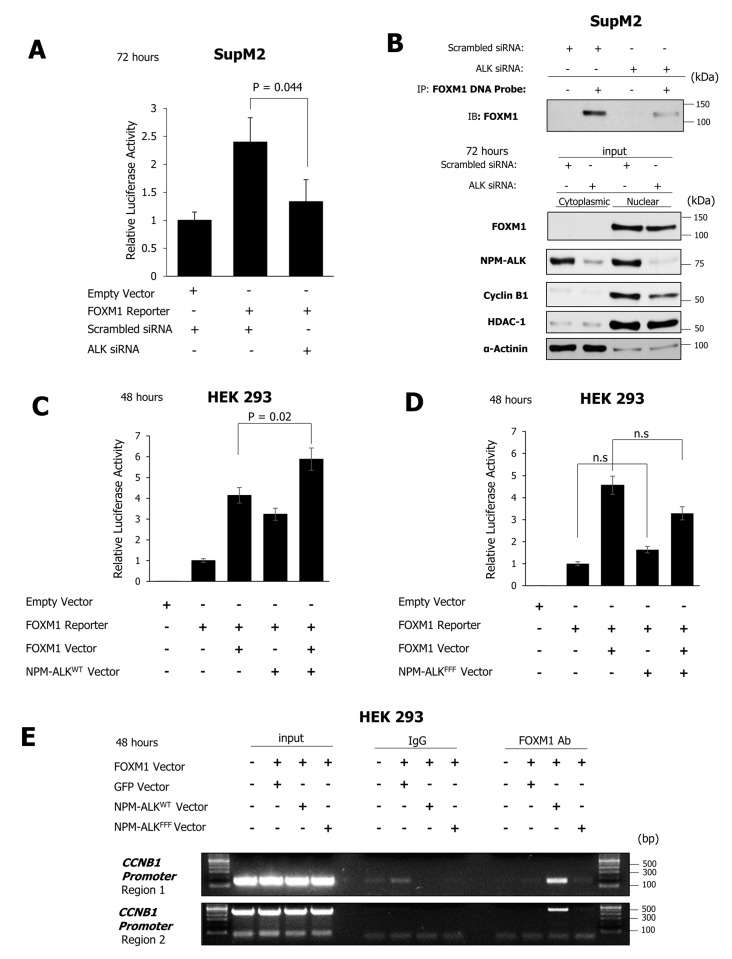
NPM-ALK promotes the transcriptional activity of FOXM1. (**A**) Cells co-transfected with siRNA to ALK or a scrambled sequence in the presence or absence of a FOXM1 consensus specific-luciferase reporter construct were assessed for luciferase activity at 72 h. (**B**) Analysis of FOXM1 binding to a FOXM1 consensus DNA sequence probe following siRNA-mediated inhibition of NPM-ALK expression at 72 h (**C**,**D**). FOXM1 luciferase reporter activity was measured at 48 h in HEK 293 cells following co-transfection of FOXM1, NPM-ALK^WT^, or NPM-ALK^FFF^ vectors. (**E**) PCR amplification of two promoter regions of CCNB1 (Cyclin B1 promoter) was performed following chromatin immunoprecipitation with a control IgG or FOXM1 antibody in HEK 293 cells, co-transfected with FOXM1, and either NPM-ALK^WT^ or kinase-dead NPM-ALK^FFF^ expression vectors for 48 h. Data are representative of two biological replicates. n.s: not significant.

To determine whether NPM-ALK plays a role in regulating the transcriptional activity of FOXM1, a FOXM1 consensus sequence-luciferase reporter construct was transiently transfected into SupM2 cells. Following siRNA-mediated downregulation of NPM-ALK, luciferase activity was abrogated, which suggests that NPM-ALK is required for FOXM1 to function as a transcription factor (Figure 6A). In keeping with this concept, siRNA-induced downregulation of NPM-ALK expression in SupM2 cells resulted in a dramatic decrease in FOXM1 protein bound to a biotinylated DNA probe containing FOXM1 consensus sequences, while the total FOXM1 protein level in the cell lysate (i.e., the input) was largely unaltered (Figure 6B). In the same experiment, Western blot studies showed that siRNA-mediated inhibition of NPM-ALK expression led to a substantial decrease in Cyclin B1, which is a well-known target of FOXM1 transcriptional activity [22]. Taken together, these data suggest that NPM-ALK is necessary for FOXM1 to exert its transcriptional regulatory activity in NPM-ALK + ALCL cells.

In order to determine whether the effect of NPM-ALK on the transcriptional activity of FOXM1 is dependent on the kinase activity of NPM-ALK, HEK 293 cells were transfected with a FOXM1 luciferase reporter construct together with either wild-type NPM-ALK or a kinase-dead mutant form of NPM-ALK [23]. As shown in Figure 6C,D, transfection of wild-type NPM-ALK (NPM-ALK^WT^), but not the kinase-dead form (NPM-ALK^FFF^), led to a significant increase in luciferase activity in the presence of exogenous FOXM1 (*p* = 0.02). In addition, FOXM1 Chromatin Immunoprecipitation (ChIP) in the presence of NPM-ALK^WT^ but not the kinase-dead variant, NPM-ALK^FFF^, showed binding of exogenous FOXM1 to Cyclin B1 (CCNB1) promoter regions (Figure 6E). These data suggest that the kinase activity of NPM-ALK is important in facilitating the transcriptional activity of FOXM1.

### 2.6. The NPM-ALK—FOXM1 Binding Is Mediated via NPM1

**Figure 7:**
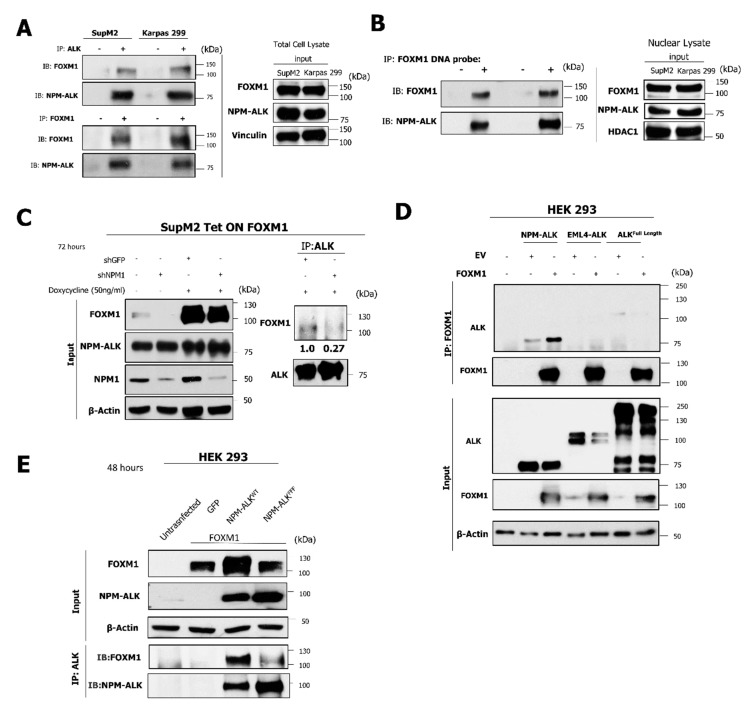
FOXM1 and NPM-ALK form a complex through their associations with NPM1. (**A**) Reciprocal co-immunoprecipitation (IP) experiments shown in the top and middle panels of FOXM1 and NPM-ALK, respectively, in the indicated cell lines. (**B**) A biotinylated DNA probe consisting of FOXM1 consensus sequences (3X) was immunoprecipitated from nuclear cell lysates of the SUPM2 and Karpas-299 cell lines, respectively. (**C**) Immunopreicpitation of NPM-ALK from SupM2 FOXM1 Tet-ON cells after treatment with doxycycline following shRNA mediated knockdown of NPM1. (**D**) Co-immunoprecipitation was performed with the FOXM1 antibody in HEK 293 cells following transfection of cells with FOXM1 and with either NPM-ALK, EML4-ALK, or ALK (full length) expression vectors. (**E**) HEK 293 cells were transfected with FOXM1 and either GFP, NPM-ALK^WT^, or NPM-ALK^FFF^ expression vectors followed by co-immunoprecipitation with a FOXM1 antibody.

In order to delineate the mechanism by which NPM-ALK facilitates the transcriptional activity of FOXM1, co-immunoprecipitation was conducted to reveal that FOXM1 complexes with NPM-ALK in SupM2 and Karpas 299 cells (Figure 7A). Furthermore, NPM-ALK was detected to be bound to a biotinylated DNA probe containing FOXM1 consensus sequences when expressed in NPM-ALK + ALCL cell lines, as was FOXM1 (Figure 7B). Taken together, our results are consistent with the hypothesis that formation of a complex involving NPM-ALK and FOXM1 is critical for FOXM1 to carry-out its transcriptional regulatory function.

In the nuclei of NPM-ALK + ALCL cells, it has been previously published that NPM-ALK is present predominantly as NPM-ALK—NPM1 heterodimers [24]. Given that, in acute myeloid leukemia, NPM1 physically binds to FOXM1 [25] via the portion of NPM1 that is not retained in the NPM-ALK fusion [24]. shRNA was employed to inhibit expression of wild-type NPM1. As predicted, a decrease in co-immunoprecipitation of NPM-ALK and FOXM1 was observed (Figure 7C). In order to confirm that binding is mediated via wild type NPM1 and the NPM portion of NPM-ALK, HEK 293 cells were transfected to express variant ALK fusion proteins including NPM-ALK, EML4-ALK, and full length wild-type ALK. In keeping with the proposed model, only NPM-ALK co-immunoprecipitated with FOXM1 (Figure 7D).

**Figure 8:**
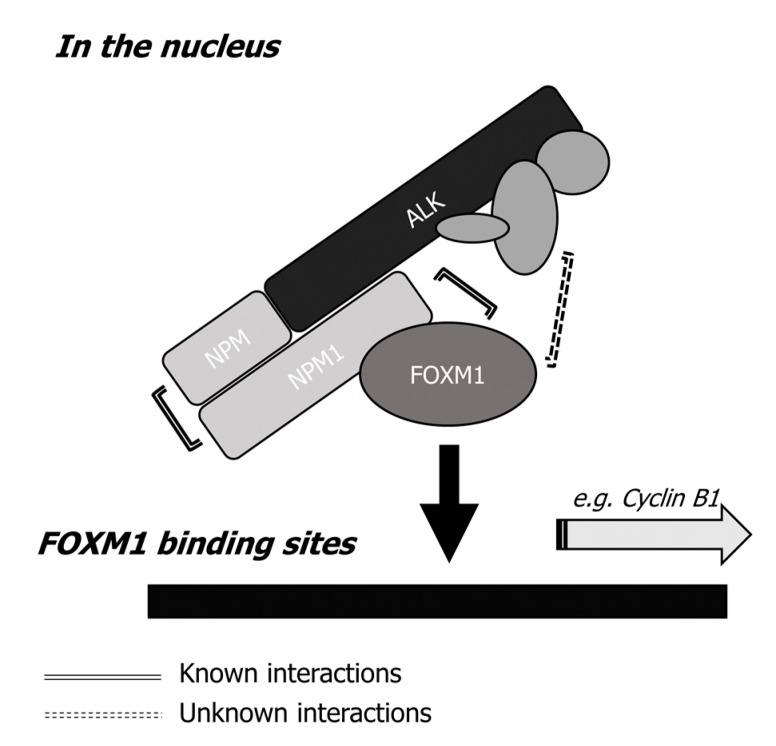
Diagram showing the binding interaction between NPM-ALK and FOXM1. NPM-ALK binds to FOXM1 through mutual interactions with a segment of NPM1, which is not represented in the NPM-ALK fusion protein. The phosphorylation of NPM-ALK further potentiates its ability to bind to FOXM1 and influence FOXM1 transcriptional activity. One possible mechanism is that phosphorylated NPM-ALK is bound by a host of other proteins, which may promote the binding between NPM-ALK and FOXM1.

As shown in Figure 7E, phosphorylation and activation of NPM-ALK is required to facilitate the DNA binding and transcriptional activity of FOXM1. In order to determine whether this activity is also required to enable the formation of a complex involving NPM1 and FOXM1, HEK293 cells were transfected to express wild-type NPM-ALK (NPM-ALK^WT^) or kinase-dead NPM-ALK (NPM-ALK^FFF^). In keeping with the data presented above, FOXM1 was found to form a complex with NPM-ALK only in the presence of wild-type NPM-ALK. Taken together, these data suggest that NPM-ALK facilitates the DNA binding and transcriptional activity of FOXM1. This process requires the phosphorylation of NPM-ALK as well as the presence of NPM1, as summarized in Figure 8.

## 3. Discussion

In solid tumors, FOXM1 has been extensively studied, and it is known to contribute to the initiation, proliferation, tumorigenicity, angiogenesis, chemo-resistance, metastatic capabilities, and stemness of malignancies [1,4]. It is believed that FOXM1 mediates its oncogenic effects via a number of molecular mechanisms, including promotion of the nuclear translocation of β-catenin [26], upregulation of a host of cell-cycle facilitators [17], increased expression of stem cell-related proteins (e.g., SOX2 and MYC) [27], inhibition of tumor suppressors including p53 [28], and increased level of vascular endothelial growth factor (VEGF) to promote angiogenesis [29]. In comparison, the biological significance of FOXM1 in hematologic malignancies is less well understood. In an early study, it was found that FOXM1 promoted the growth of thymic lymphoma in a p53-null mouse model [30]. In another study, conditional knockdown of FOXM1 in precursor B-cell lymphoblastic leukemia cell lines was found to significantly prolong the survival of mice xenografted with leukemic cells [5]. In a study of plasma cell myeloma, overexpression of FOXM1 in cell lines resulted in a significantly higher tumor volume of xenografts formed in mice [8]. A high level of FOXM1 expression detectable by immunohistochemistry was found in ~85% cases of diffuse large B-cell lymphoma, and pharmacologic inhibition of FOXM1 in lymphoma cell lines substantially decreased invasiveness in vitro, which correlated with a reduction in expression of Ki-67 and two epithelial-to-mesenchymal transition markers (MMP-2 and MMP-9) [31]. Lastly, in two studies of acute myeloid leukemia, the silencing and reduction of FOXM1 led to decreased tumorigenic properties in both in vitro and in vivo models [10,32].

The expression of FOXM1 in NPM-ALK+ ALCL, whose normal counterpart is believed to be mature T-cells [33,34], is an aberrant event. In this regard, it has been shown that the expression of FOXM1 mRNA is tightly regulated during T-cell development, with the expression of FOXM1 first detectable in double-negative (i.e., negative for CD4 and CD8) thymocytes, which reaches its peak at the stage of immature double positive (i.e., positive for both CD4 and CD8) thymocytes, dramatically decreasing on becoming single-positive (e.g., positive for CD4 or CD8) thymocytes, and becoming undetectable in fully mature lymphocytes in the periphery [35]. In keeping with these findings, we found that PBMC cells, which include mature T-cells, had no detectable FOXM1 protein. Based on these observations, it is possible that the normal mechanism that is responsible for ’silencing’ FOXM1 protein expression at the mature T-cell stage has become defective during the oncogenesis of NPM-ALK + ALCL.

In support of the concept that FOXM1 is transcriptionally active in NPM-ALK + ALCL, FOXM1 was found in this study to be localized mostly in the nuclei of NPM-ALK + ALCL cells. Furthermore, the FOXM1 detectable in NPM-ALK+ cell lines bound to a DNA probe carrying its consensus sequences and showed luciferase activity upon transfection of a FOXM1 luciferase reporter construct. Experimental manipulation of the expression level of FOXM1 resulted in the expected changes in Cyclin B1, which is a well-known FOXM1 downstream target [22]. Moreover, FOXM1 contributes to the activation/phosphorylation of NPM-ALK. Similarly, the activation/phosphorylation level of STAT3 mirrored that of pALK. The exact mechanism by which FOXM1 contributes to increased pALK and pSTAT3 levels remain a mystery. In this regard, it is known that FOXM1 can regulate several cellular signaling pathways through multiple mechanisms. For example, it has been shown that FOXM1 upregulates the expression and activity of STAT3 in a β-catenin-dependent manner in glioblastoma cells [36]. In osteosarcoma cells, FOXM1 was also known to transcriptionally regulate the expression and phosphorylation c-Jun N-terminal kinase (JNK1) to drive cell proliferation [37].

One of the key findings of this study is that NPM-ALK promotes the DNA binding and transcriptional activity of FOXM1. This conclusion is based on the observations that knockdown of NPM-ALK significantly reduced FOXM1 luciferase reporter activity and its binding to a DNA probe carrying FOXM1 consensus sequences. Furthermore, results from the ChIP assay provided further support and validation to this conclusion. The functional role of NPM-ALK localized to the nucleus is largely unknown. The data presented in this case, considering that FOXM1 is largely localized to the nucleus, suggest that NPM-ALK can exert oncogenic effects in the nuclei of NPM-ALK + ALCL cells by promoting the biological activity of oncogenic transcription factors, such as FOXM1.

Our experimental results also have shed light into how NPM-ALK regulates the transcriptional activity of FOXM1. Specifically, our data point to a model in which the NPM-ALK facilitated DNA binding (and, hence, the transcriptional activity) of FOXM1 requires physical interaction between NPM-ALK and FOXM1. How exactly FOXM1 binds to NPM-ALK was thoroughly examined in this study. Two important features of NPM-ALK localized in the nucleus of NPM-ALK + ALCL cells are highly relevant. First, NPM-ALK has been reported to exist predominantly as NPM-ALK—NPM1 heterodimer in the nuclei of NPM-ALK + ALCL cells [24]. Second, a previously published study showed that FOXM1 can bind to the heterodimer domain of NPM1 [38], which is a segment close to the C-terminus of NPM1. This is not represented in the fusion NPM-ALK protein [24,39]. Our experimental data is consistent with the hypothetical model depicted in Figure 8. We found that the NPM1 portion of NPM-ALK is crucial to binding to FOXM1, as EML-ALK [40] and full length ALK [41] were unable to bind to FOXM1. In this regard, our data suggest that the ALK portion alone cannot efficiently interact with FOXM1. Thus, we found further evidence of the pathogenetic role of NPM1, which is a protein highly implicated in the pathogenesis of acute myeloid leukemias (AML), in NPM-ALK + ALCL [42]. This is in parallel to how NPM1 is believed to promote tumorigenesis. Specifically, NPM1 has been shown to facilitate DNA binding and transcriptional activity of oncoproteins such as c-Myc [43] and NF-κB-p65 [44].

It appears that the phosphorylation status of NPM-ALK is important in regulating the biological activity of FOXM1. This conclusion is based on the observation that transfection of NPM-ALK^WT^, but not the kinase-dead NPM-ALK^FFF^, significantly promoted the transcription activity of FOXM1. This is rather intriguing, as it has been reported that most NPM-ALK proteins present in the nuclei of NPM-ALK + ALCL cells exist in the form of NPM-ALK—NPM1, which is not as highly phosphorylated as the NPM-ALK dimer present in the cytoplasm of these cells [39]. Nonetheless, a weak but definitive level of phosphorylated ALK can be detected in the nuclear fraction of NPM-ALK + ALCL [39]. It could be speculated that this phosphorylation occurs through the presence of other kinases present in the nucleus of ALK + ALCL. It is possible that this relatively low level of NPM-ALK phosphorylation may have resulted in an optimal three-dimensional conformation in the NPM-ALK—NPM1 dimer, which facilitates the physical interaction of NPM1 and FOXM1. This low level of NPM-ALK phosphorylation may have attracted the binding of other proteins to the NPM-ALK—NPM1:FOXM1 complex, which facilitates the DNA binding of FOXM1.

## 4. Materials and Methods

### 4.1. Cell Lines and Materials

All NPM-ALK + ALCL and Jurkat cells were cultured in RPMI 1640 medium (Invitrogen, Carlsbad, CA, USA). HEK 293 and 293T cells were maintained in high glucose DMEM. Media were supplemented with 10% fetal bovine serum and 1% penicillin and streptomycin (Invitrogen). All doxycycline inducible stable cell lines were maintained in 10% tetracycline approved fetal bovine serum (Clonetech, Mountain View, CA, USA), 1% penicillin and streptomycin, and 0.5 µg/mL puromycin (Sigma-Aldrich, St. Louis, MO, USA). All cells were maintained in a 5% CO2 atmosphere at 37 °C. Thiostrepton and doxorubicin were purchased from Sigma-Aldrich.

### 4.2. Immunohistochemistry

Immunohistochemical analysis was performed as described previously [45]. For the 21 NPM-ALK + ALCL patient tumor sections, an anti-FOXM1 rabbit antibody (1:500, Abcam, Cambridge, United Kingdom; #207298) was used. For NPM-ALK+ ALCL transgenic mouse sections, an anti-FOXM1 mouse antibody (1:500, Santa Cruz (SC), Dallas, TX, USA; #376471) was used. Heat induced epitope retrieval was performed by microwaving in Tris-EDTA buffer, pH 9.0, for 20 min. Reactive human tonsil sections were used as a negative control.

### 4.3. Cell Viability

The CellTiter 96^®^ AQueous One Solution Cell Assay (i.e MTS assay) (Promega, Madison, WI, USA) was used to measure cell viability following drug treatments. The trypan blue exclusion assay (Amresco, Solon, OH, USA) was used to measure cell viability for time-dependent experiments.

### 4.4. Reverse Transcriptase Polymerase Chain Reaction (RT-PCR) and Quantitative Real Time PCR (qPCR)

Total RNA was extracted from cell lines with the RNeasy Plus Mini Kit (Qiagen, Valencia, CA, USA). Reverse Transcription (RT) reactions were performed with 1 μg of total RNA using the Superscript First-Strand Synthesis System Kits (Invitrogen). RT-PCR parameters included an initial denaturing step at 95 °C for 10 min followed by 30 cycles at 95 °C for 45 s, annealing at 56 °C for 30 s, and extension at 72 °C for 30 s, which was followed by a final extension step at 72 °C for 10 min. PCR products were then analyzed by 2% agarose gel electrophoresis. Quantitative real-time PCR reactions were performed as described previously [46]. The qPCR cycle was as follows: denaturation at 95 °C for 10 min, followed by 40 cycles at 95 °C for 15 s, annealing and extension at 60 °C for 1 min. All target genes were normalized to glyceraldehyde-3-phosphate dehydrogenase (GAPDH) expression levels. The relative expression was determined using the ΔΔ−CT method. Table S1 contains the list of primer sequences used for this study.

### 4.5. Plasmids and siRNA

Short hairpin RNA (shRNA) plasmids for FOXM1 and NPM1 (pLKO.1), and short interfering RNA (siRNA) for ALK were all purchased from Dharmacon (Lafayette, CO, USA). The GFP shRNA plasmid was purchased from Addgene (Watertown, MA, USA) (#30323). Vectors for doxycycline inducible overexpression of FOXM1B/C vectors were made with the pCW57.1 vector (Addgene 68811 and 68810) [47]. The empty vector luciferase reporter and FOXM1 luciferase reporter containing FOXM1 consensus sequences were based on the pGL4.10 backbone [48]. The NPM-ALK (pcDNA3.1) plasmid was a gift from Dr. Stephan Morris. NPM-ALK^WT^ and NPM-ALK^FFF^ (HB tagged) expression vectors have been previously characterized [23].

### 4.6. Transient Transfections

The electro square electroporator BTX ECM 800 (225 V, 8.5 milliseconds, 6 pulses) was used for transient transfections of ALK + ALCL cell lines with siRNAs and plasmid vectors. Short interfering RNAs (siRNAs) for ALK and scrambled siRNA were purchased from Dharmacon. Per 10 million ALK + ALCL cells, 1 nanomole of siRNAs were used. Western blots were used to determine the efficiency of the knockdown. Transient transfections of 293T cells were performed in six-well plates. Additionally, 1.5 µg of plasmid or 200 picomole of siRNA were combined with 5 µL Lipofectamine and added to each well. Cells were harvested 24 h following transfection.

### 4.7. Lentiviral Based Transduction

A lentivirus-based transduction strategy optimized for T-lymphocytes was used to stably transfect ALK + ALCL cell lines [49]. Lentivirus production was achieved in 100 mm dishes by transfecting 293T cells with 9 µg transfer vector, 9 µg psPax2, and 3 µg pMD2.G plasmids (Addgene) using 30 µL Lipofectamine (Life Technologies, Carlsbad, CA, USA). After 48 h, the viral supernatant was collected and filtered before 2 million ALK + ALCL cells were incubated with 1 mL of viral supernatant containing 0.8 µg/mL polybrene (Sigma-Aldrich) in a six-well plate. The plate was then spun at 1000× *g* and 32 °C at 2 h to facilitate viral infection. Following the spin-infection step, 1 mL of fresh medium was added to each well. To increase the transduction efficiency, the spin-infection step was repeated the next day with a fresh virus supernatant. In addition, 24 h after the second infection, cells were washed twice with cold PBS and resuspended in fresh medium. Cells were then collected for subsequent assays and Western blot analysis. For the development of monoclonal stable cell lines, cells were selected with increasing concentrations of puromycin, increasing from 0.5 µg/mL to 3 µg/mL followed by single cell selection by serial dilution.

### 4.8. Flow Cytometric Analyses

The CellTrace™ CFSE Cell Proliferation Kit was purchased from Thermo Fisher Scientific (Carlsbad, CA, USA). In addition, 5 nmol of CFSE (carboxyfluorescein succinimidyl ester) dye (in DMSO) was incubated with 1 million cells for 30 min. Flow cytometry was conducted, and viable cells were gated using forward and side scatter parameters. Over the next three days, 200,000 cells were analyzed each day for loss of CFSE (Carboxyfluorescein succinimidyl ester) staining. To determine apoptosis and conduct cell cycle analysis of NPM-ALK + ALCL cell lines, cells were stained with the FITC Annexin V Apoptosis Detection Kit I (BD Biosciences, Franklin Lakes, NJ, USA), according to the manufacturer’s recommendations. Annexin V positivity was analyzed with BD FACSDiva software. Flow cytometry experimentation results are representative of two or more biological replicates.

### 4.9. Soft Agar Colony Formation Assay

The soft agar colony formation assay was performed as described in a prior study [50]. Following experimental treatments, 10,000 cells were seeded in six-well plates in triplicates. Colonies were grown for two weeks, which was followed by staining of the plates with 0.05% crystal violet (Sigma-Aldrich) and subsequent imaging.

### 4.10. Co-Immunoprecipitation and Western Blotting

Co-immunoprecipitation and Western blotting were performed as described previously [16]. Primary antibodies used included: anti-FOXM1 (1:500, Santa Cruz (SC), #271746, 1:500, Abcam, #207298), anti-pALK Y1604 (1:500, Cell Signaling technologies (CST), #3341S), anti-ALK (1:1000, CST, #3633), anti-pSTAT3 (Y705) (1:2000, CST, #9145), anti-STAT3 (1:1000, CST, #124H6), anti-Cyclin B1 (1:1000, SC, #752), anti-β-Actin (1:8000, SC, #47778), anti-PARP (1:1000, CST, #9542), anti-Caspase 3(1:1000, CST, #9662), anti-Survivin (1:1000, CST, #2808), anti-NPM1 (1:2000, Milipore Sigma, clone 3C9), anti-Vinculin (1:500, SC, #25336), anti HDAC-1 (1:500, SC, #81598), and anti-α-Actinin (1:500, SC, 17829). Secondary antibodies used were HRP-conjugated anti-mouse (1:2000, CST, #7076) and anti-rabbit (1:2000, CST, #7074). Detailed information of western blot can be found at Figure S5.

### 4.11. FOXM1 DNA Probe Pulldown

Cells were washed with cold phosphate buffered saline (PBS) and then separated into cytoplasmic and nuclear fractions using the Pierce NE-PER kit (Fisher Scientific Canada, Ottawa, ON, Canada), according to the manufacturer’s instructions. As per a previous study [51], 400 µg nuclear protein was incubated with or without 300 pmol of a 5′ biotin-labeled probe (Integrated DNA Technologies, Edmonton, AB) containing FOXM1 consensus sequences. The sequence of the FOXM1 probe with the consensus site underlined is as follows: AAACAAACAAACAATCAAACAA. Mutant DNA and unbiotinylated DNA probes were used as negative controls to optimize the protocol. Following addition of the biotinylated probe, the mixture was then incubated with rotation for 30 min at room temperature. Streptavidin agarose beads (50 μL, Fisher Scientific) were then added to each sample, which were then incubated with rotation overnight at 4 °C. The following day, the samples were collected by centrifugation at low speed and the supernatant was discarded. The beads were then washed three times with ice cold PBS. Protein elution was achieved by boiling the beads at 100 °C in a 4× sample loading buffer, before loading proteins onto SDS-PAGE gels.

### 4.12. Luciferase Assay

The luciferase reporter assay was performed using a Luciferase Assay System kit (Promega, Corporation, Madison, USA), according to the manufacturer’s protocol. In brief, cells were transiently transfected with either the empty or FOXM1 luciferase reporter, as mentioned above. Cells were lysed with a passive lysis buffer followed by estimation of protein concentration. Equal amounts of protein for each sample were then assessed for luciferase activity using the FLUOstar Omega multi-mode microplate reader (BMG Labtech, Ortenburg, Germany) to read and analyze data.

### 4.13. Chromatin Immunoprecipitation

Chromatin Immunoprecipitation (ChIP) was performed according to the manufacturer’s instructions (Upstate-Millipore Chromatin Immunoprecipitation Assay Kit (Temecula, CA, USA)). Human Embryonic Kidney (HEK) 293 cells were plated in 15-centimeter plates. Transient transfections in HEK 293, as mentioned above, were performed with GFP, FOXM1, NPM-ALK^WT^, and NPM-ALK^FFF^ cDNA-containing expression vectors for 48 h. At approximately 90% confluency, cells were fixed with 1% formaldehyde (in culture media) for 20 min at room temperature with rocking. Formaldehyde was then quenched by the addition of glycine and incubated for an additional 10 min. Cells were collected by centrifugation, and washed and lysed with SDS lysis buffer (1% SDS, 10 mM EDTA). Chromatin was sheared by sonication on ice using a Fisher Scientific Sonic Dismembrator Ultrasonic Processor Model 705 (Fisher Scientific) at 25% power with 10 pulses of 20 s sonication and 30 s rest to give optimized DNA fragments of between 200 bp and 1 kb. Chromatin was subsequently suspended in ChIP Dilution Buffer and incubated with 5 μg of either normal rabbit IgG (Cell Signaling) or anti-FOXM1 antibody (Santa Cruz) overnight at 4 °C. Next, Protein A agarose beads were added to the chromatin/FOXM1 or rabbit IgG antibody for an additional 6 h at 4 °C. The immunoprecipitated material bound to agarose beads was then washed once with a low salt wash buffer, a high salt wash buffer, LiCl wash buffer, and then twice in TE buffer. To elute the immunoprecipitated material from the beads, the bead-bound chromatin was incubated with ChIP Elution Buffer. Input chromatin and DNA/protein formaldehyde crosslinks were then reversed with 5 M NaCl at 65 °C overnight. Proteins were digested with Proteinase K (Ambion, Invitrogen, Burlington, ON, Canada) at 55 °C for 2 h and DNA was purified using a PCR Purification kit (Qiagen). The resulting ChIP DNA and input DNA were then amplified by PCR with previously validated *CCNB1* ChIP PCR primers [19,52] (see Table S1).

### 4.14. Statistical Analysis

Numerical data have been expressed as the mean ± standard deviation obtained from the number of replicates mentioned in the figure legends. The two-tailed Student’s *t* test and one-way ANOVA was used to determine the significance with α = 0.05. Statistical analysis was performed with GraphPad software (La Jolla, CA, USA).

## 5. Conclusions

In conclusion, our study reveals, for the first time, the importance of FOXM1 in NPM-ALK + ALCL. The oncogenic effects of FOXM1 appear to be closely linked to NPM-ALK. This association contributes to the oncogenesis in these tumors. Furthermore, FOXM1 may be a potential therapeutic target in ALK + ALCL, and disruption of the binding between FOXM1 and NPM1 in the NPM-ALK—NPM1 heterodimers may serve as a highly specific anti-cancer therapeutic approach for NPM-ALK + ALCL.

## Supplementary Materials

The following are available online at https://www.mdpi.com/2072-6694/11/8/1119/s1, Figure S1: Downregulation of FOXM1 induces apoptosis, Figure S2: FOXM1 shRNA significantly inhibits cell division in UCONN-L2, Figure S3: shRNA knockdown of FOXM1 largely abrogates soft agar colony formation in UCONN-L2 cells, Figure S4: Overexpression of FOXM1 provides resistance to thiostrepton mediated growth inhibition of FOXM1 in a tetracycline inducible FOXM1 SupM2 cell line, Figure S5: Uncropped western blot films, Table S1: PCR primer sets used in the study.

## References

[1] Koo C.-Y., Muir K.W., Lam E.W.-F. (2012). FOXM1: From cancer initiation to progression and treatment. Biochim. Biophys. Acta (BBA) Gene Regul. Mech..

[2] Korver W., Roose J., Clevers H. (1997). The winged-helix transcription factor Trident is expressed in cycling cells. Nucleic Acids Res..

[3] Chen X., Muller G.A., Quaas M., Fischer M., Han N., Stutchbury B., Sharrocks A.D., Engeland K. (2013). The forkhead transcription factor FOXM1 controls cell cycle-dependent gene expression through an atypical chromatin binding mechanism. Mol. Cell. Biol..

[4] Myatt S.S., Lam E.W.-F. (2007). The emerging roles of forkhead box (Fox) proteins in cancer. Nat. Rev. Cancer.

[5] Buchner M., Park E., Geng H.M., Klemm L., Flach J., Passegue E., Schjerven H., Melnick A., Paietta E., Kopanja D. (2015). Identification of FOXM1 as a therapeutic target in B-cell lineage acute lymphoblastic leukaemia. Nat. Commun..

[6] Tufekci O., Yandim M.K., Oren H., Irken G., Baran Y. (2013). Targeting FOXM1 Transcription Factor In T-Cell Acute Lymphoblastic Leukemia. Blood.

[7] Wang J.Y., Jia X.H., Xing H.Y., Li Y.J., Fan W.W., Li N., Xie S.Y. (2015). Inhibition of Forkhead box protein M1 by thiostrepton increases chemosensitivity to doxorubicin in T-cell acute lymphoblastic leukemia. Mol. Med. Rep..

[8] Gu C., Yang Y., Sompallae R., Xu H., Tompkins V.S., Holman C., Hose D., Goldschmidt H., Tricot G., Zhan F. (2016). FOXM1 is a therapeutic target for high-risk multiple myeloma. Leukemia.

[9] Uddin S., Hussain A.R., Ahmed M., Siddiqui K., Al-Dayel F., Bavi P., Al-Kuraya K.S. (2012). Overexpression of FoxM1 offers a promising therapeutic target in diffuse large B-cell lymphoma. Haematologica.

[10] Khan I., Patel A.A., Halasi M., Schultz R., Chen Y.H., Aardsma N., Kalakota N., Liu L.C., Mahmud N., Gann P. (2017). Validation of FOXM1 As a Therapeutic Target in Acute Myeloid Leukemia. Blood.

[11] Amin H.M., Lai R. (2007). Pathobiology of ALK+ anaplastic large-cell lymphoma. Blood.

[12] Zamo A., Chiarle R., Piva R., Howes J., Fan Y., Chilosi M., Levy D.E., Inghirami G. (2002). Anaplastic lymphoma kinase (ALK) activates Stat3 and protects hematopoietic cells from cell death. Oncogene.

[13] Slupianek A., Nieborowska-Skorska M., Hoser G., Morrione A., Majewski M., Xue L., Morris S.W., Wasik M.A., Skorski T. (2001). Role of phosphatidylinositol 3-kinase-Akt pathway in nucleophosmin/anaplastic lymphoma kinase-mediated lymphomagenesis. Cancer Res..

[14] Marzec M., Kasprzycka M., Liu X., Raghunath P.N., Wlodarski P., Wasik M.A. (2006). Oncogenic tyrosine kinase NPM/ALK induces activation of the MEK/ERK signaling pathway independently of c-Raf. Oncogene.

[15] Werner M.T., Zhao C., Zhang Q., Wasik M.A. (2017). Nucleophosmin-anaplastic lymphoma kinase: The ultimate oncogene and therapeutic target. Blood.

[16] Gelebart P., Hegazy S.A., Wang P., Bone K.M., Anand M., Sharon D., Hitt M., Pearson J.D., Ingham R.J., Ma Y. (2012). Aberrant expression and biological significance of Sox2, an embryonic stem cell transcriptional factor, in ALK-positive anaplastic large cell lymphoma. Blood Cancer J..

[17] Wierstra I. (2013). The Transcription Factor FOXM1 (Forkhead box M1): Proliferation-Specific Expression, Transcription Factor Function, Target Genes, Mouse Models, and Normal Biological Roles. Adv. Cancer Res..

[18] Tufekci O., Yandim M.K., Oren H., Irken G., Baran Y. (2015). Targeting FoxM1 transcription factor in T-cell acute lymphoblastic leukemia cell line. Leuk. Res..

[19] Ma R.Y.M., Tong T.H.K., Cheung A.M.S., Tsang A.C.C., Leung W.Y., Yao K.-M. (2005). Raf/MEK/MAPK signaling stimulates the nuclear translocation and transactivating activity of FOXM1c. J. Cell Sci..

[20] Malcolm T.I.M., Villarese P., Fairbairn C.J., Lamant L., Trinquand A., Hook C.E., Burke G.A.A., Brugières L., Hughes K., Payet D. (2016). Anaplastic large cell lymphoma arises in thymocytes and requires transient TCR expression for thymic egress. Nat. Commun..

[21] Hegde N.S., Sanders D.A., Rodriguez R., Balasubramanian S. (2011). The transcription factor FOXM1 is a cellular target of the natural product thiostrepton. Nat. Chem..

[22] Leung T.W.C., Lin S.S.W., Tsang A.C.C., Tong C.S.W., Ching J.C.Y., Leung W.Y., Gimlich R., Wong G.G., Yao K.M. (2001). Over-expression of FoxM1 stimulates cyclin B1 expression. FEBS Lett..

[23] Wang P., Wu F., Zhang J., McMullen T., Young L.C., Ingham R.J., Li L., Lai R. (2010). Serine phosphorylation of NPM–ALK, which is dependent on the auto-activation of the kinase activation loop, contributes to its oncogenic potential. Carcinogenesis.

[24] Box J.K., Paquet N., Adams M.N., Boucher D., Bolderson E., O’Byrne K.J., Richard D.J. (2016). Nucleophosmin: From structure and function to disease development. BMC Mol. Biol..

[25] Bhat U.G., Jagadeeswaran R., Halasi M., Gartel A.L. (2011). Nucleophosmin interacts with FOXM1 and modulates the level and localization of FOXM1 in human cancer cells. J. Biol. Chem..

[26] Zhang N., Wei P., Gong A.H., Chiu W.T., Lee H.T., Colman H., Huang H., Xue J.F., Liu M.G., Wang Y. (2011). FoxM1 promotes beta-catenin nuclear localization and controls wnt target-gene expression and glioma tumorigenesis. Cancer Cell.

[27] Xie Z.Q., Tan G.X., Ding M.A., Dong D.F., Chen T.H., Meng X.X., Huang X.Q., Tan Y.J. (2010). Foxm1 transcription factor is required for maintenance of pluripotency of P19 embryonal carcinoma cells. Nucleic Acids Res..

[28] Pandit B., Halasi M., Gartel A.L. (2009). P53 negatively regulates expression of FoxM1. Cell Cycle.

[29] Ahmad A., Wang Z., Kong D., Ali S., Li Y., Banerjee S., Ali R., Sarkar F.H. (2010). FoxM1 down-regulation leads to inhibition of proliferation, migration and invasion of breast cancer cells through the modulation of extra-cellular matrix degrading factors. Breast Cancer Res. Treat..

[30] Wang Z.B., Zheng Y., Park H.J., Li J., Carr J.R., Chen Y.J., Kiefer M.M., Kopanja D., Bagchi S., Tyner A.L. (2013). Targeting FoxM1 effectively retards p53-null lymphoma and sarcoma. Mol. Cancer Ther..

[31] Siraj A.K., Hussain A.R., Ahmed M., Ahmed S.O., Bu R., Al-Dayel F., Bavi P., Uddin S., Al-Kuraya K.S. (2011). FoxM1 expression and its association with matrix metalloproteinases in diffuse large B-cell lymphoma. Cancer Res..

[32] Khan I., Halasi M., Patel A., Schultz R., Kalakota N., Chen Y.H., Aardsma N., Liu L., Crispino J.D., Mahmud N. (2018). FOXM1 contributes to treatment failure in acute myeloid leukemia. JCI Insight.

[33] Falini B., Bigerna B., Fizzotti M., Pulford K., Pileri S.A., Delsol G., Carbone A., Paulli M., Magrini U., Menestrina F. (1998). ALK expression defines a distinct group of T/null lymphomas (“ALK lymphomas”) with a wide morphological spectrum. Am. J. Pathol..

[34] Falini B., Martelli M.P. (2009). Anaplastic large cell lymphoma: Changes in the World Health Organization classification and perspectives for targeted therapy. Haematologica.

[35] Xue L., Chiang L., He B., Zhao Y.Y., Winoto A. (2010). FoxM1, a Forkhead Transcription Factor Is a Master Cell Cycle Regulator for Mouse Mature T Cells but Not Double Positive Thymocytes. PLoS ONE.

[36] Zhang S.C., Gong A.H., Wei P., Zhou A.D., Yao J., Yuan Y., Lang F., Rao G., Huang S.Y. (2015). FoxM1 drives a feed-forward STAT3-activation signaling loop to promote the self-renewal and tumorigenicity of glioblastoma stem cells. Cancer Res..

[37] Wang I.C., Chen Y.J., Hughes D.E., Ackerson T., Major M.L., Kalinichenko V.V., Costa R.H., Raychaudhuri P., Tyner A.L., Lau L.F. (2008). FoxM1 regulates transcription of JNK1 to promote the G(1)/S transition and tumor cell invasiveness. J. Biol. Chem..

[38] Khan I., Zia M., Halasi M., Gann P., Gaitonde S., Gartel A. (2015). FOXM1 Binds Nucleophosmin in AML and Confers Resistance to Chemotherapy. Blood.

[39] Ceccon M., Merlo M.E.B., Mologni L., Poggio T., Varesio L.M., Menotti M., Bombelli S., Rigolio R., Manazza A.D., Di Giacomo F. (2016). Excess of NPM-ALK oncogenic signaling promotes cellular apoptosis and drug dependency. Oncogene.

[40] Sabir S.R., Yeoh S., Jackson G., Bayliss R. (2017). EML4-ALK Variants: Biological and Molecular Properties, and the Implications for Patients. Cancers.

[41] Wellstein A. (2012). ALK receptor activation, ligands and therapeutic targeting in glioblastoma and in other cancers. Front. Oncol..

[42] Heath E.M., Chan S.M., Minden M.D., Murphy T., Shlush L.I., Schimmer A.D. (2017). Biological and clinical consequences of NPM1 mutations in AML. Leukemia.

[43] Li Z., Boone D., Hann S.R. (2008). Nucleophosmin interacts directly with c-Myc and controls c-Myc-induced hyperproliferation and transformation. Proc. Natl. Acad. Sci. USA.

[44] Lin J., Kato M., Nagata K., Okuwaki M. (2017). Efficient DNA binding of NF-κB requires the chaperone-like function of NPM1. Nucleic Acids Res..

[45] Gelebart P., Anand M., Armanious H., Peters A.C., Dien Bard J., Amin H.M., Lai R. (2008). Constitutive activation of the Wnt canonical pathway in mantle cell lymphoma. Blood.

[46] Wu C., Molavi O., Zhang H., Gupta N., Alshareef A., Bone K.M., Gopal K., Wu F., Lewis J.T., Douglas D.N. (2015). STAT1 is phosphorylated and downregulated by the oncogenic tyrosine kinase NPM-ALK in ALK-positive anaplastic large-cell lymphoma. Blood.

[47] Barger C.J., Zhang W., Hillman J., Stablewski A.B., Higgins M.J., Vanderhyden B.C., Odunsi K., Karpf A.R. (2015). Genetic determinants of FOXM1 overexpression in epithelial ovarian cancer and functional contribution to cell cycle progression. Oncotarget.

[48] Barger C.J., Branick C., Chee L., Karpf A.R. (2019). Pan-Cancer Analyses Reveal Genomic Features of FOXM1 Overexpression in Cancer. Cancers.

[49] Lee J., Sadelain M., Brentjens R. (2009). Retroviral transduction of murine primary T lymphocytes. Methods Mol. Biol. (Clifton N.J.).

[50] Borowicz S., Van Scoyk M., Avasarala S., Karuppusamy Rathinam M.K., Tauler J., Bikkavilli R.K., Winn R.A. (2014). The Soft Agar Colony Formation Assay. JoVE.

[51] Wu K.K., Bina M. (2006). Analysis of Protein-DNA Binding by Streptavidin-Agarose Pulldown. Gene Mapping, Discovery, and Expression: Methods and Protocols.

[52] Ketola K., Bishop J., Nip K.M., Kim S., Ladan F., Gleave M., Zoubeidi A. (2014). Inhibition of FOXM1 targets both high and low PSA expressing prostate cancer cells resistant to Enzalutamide. Cancer Res..

