# Supplementary material for: NPM-ALK Is a Key Regulator of the Oncoprotein FOXM1 in ALK-Positive Anaplastic Large Cell Lymphoma
Source: Cancers (Basel). 2019 Aug 6;11(8):1119. doi: 10.3390/cancers11081119 (PMC6721812; doi:10.3390/cancers11081119)
Supplement: Supplementary file 1 [file cancers-11-01119-s001.pdf]

Supplementary Materials

NPM-ALK Is a Key Regulator of the Oncoprotein FOXM1 in ALK-Positive Anaplastic Large Cell Lymphoma

Moinul Haque, Jing Li, Yung-Hsing Huang, Meaad Almowaled, Carter J. Barger, Adam R. Karpf, Peng Wang, Will Chen, Suzanne D. Turner and Raymond Lai

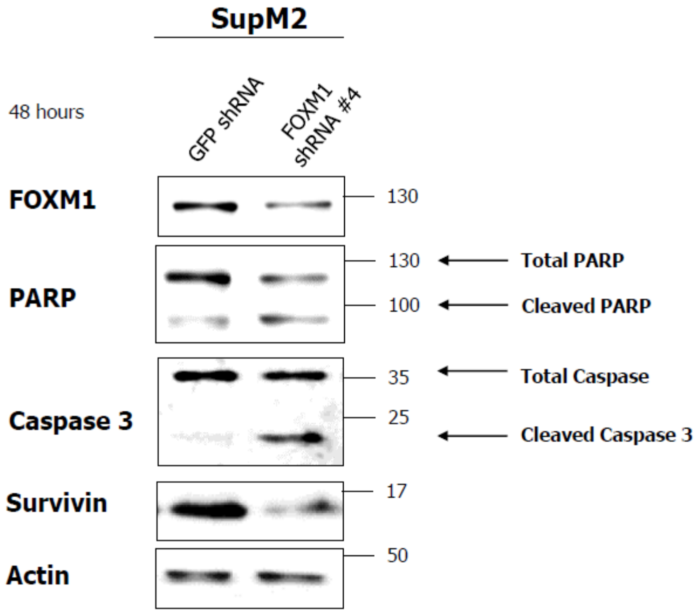

Figure S1. Downregulation of FOXM1 induces apoptosis. Western blot analysis of SupM2 cells infected with FOXM1 shRNA for 48 hours showed cleavage of PARP and caspase 3, markers of apoptosis. Moreover, the expression of an anti-apoptotic protein, Survivin, was dramatically reduced. β-actin served as the loading control. Data are representative of three biological replicates.

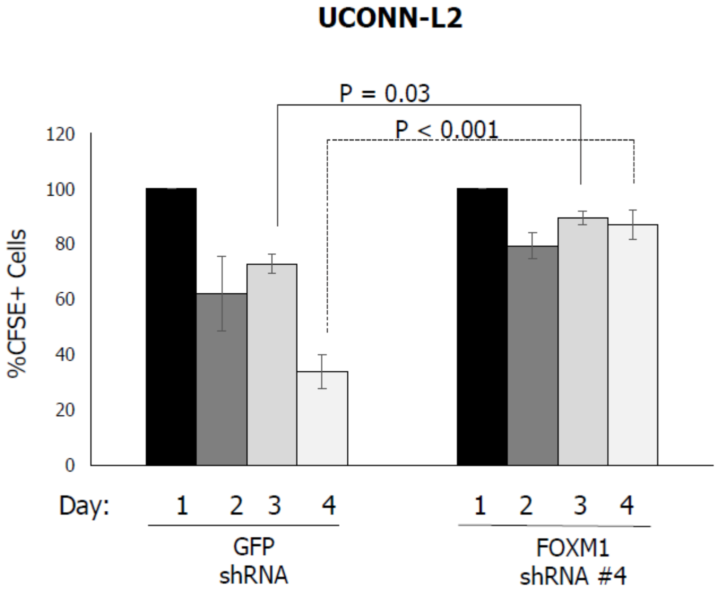

Figure S2. FOXM1 shRNA significantly inhibits cell division in UCONN-L2. Infection of UCONN-L2 with FOXM1 shRNA significantly preserved the CFSE content, as compared to cells treated with GFP shRNA on day 3 and day 4. Data are representative of two biological replicates.

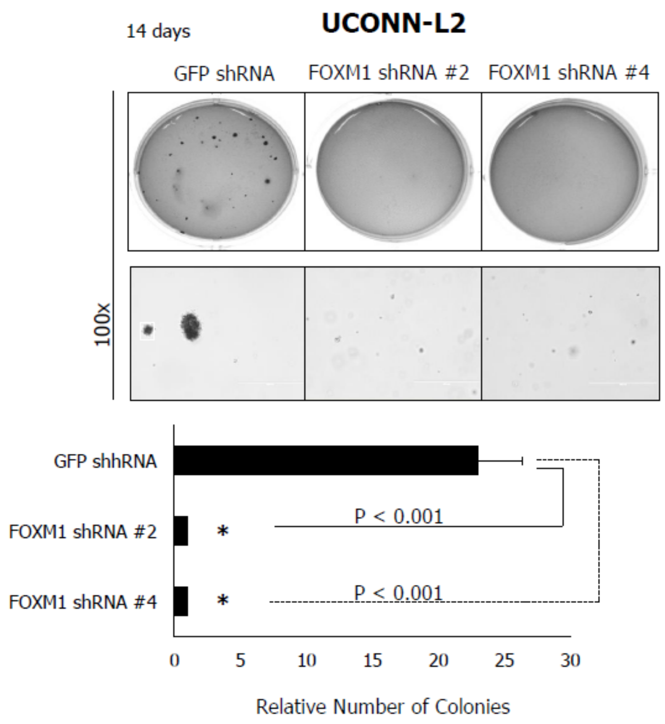

Figure S3. shRNA knockdown of FOXM1 largely abrogates soft agar colony formation in UCONN-L2 cells. Soft agar colony formation of UCONN-L2 cells infected with either GFP shRNA or FOXM1 shRNA was evaluated. The boxed area (middle right panel) shows the cut-off size for a colony to be counted. Data are representative of two biological replicates.

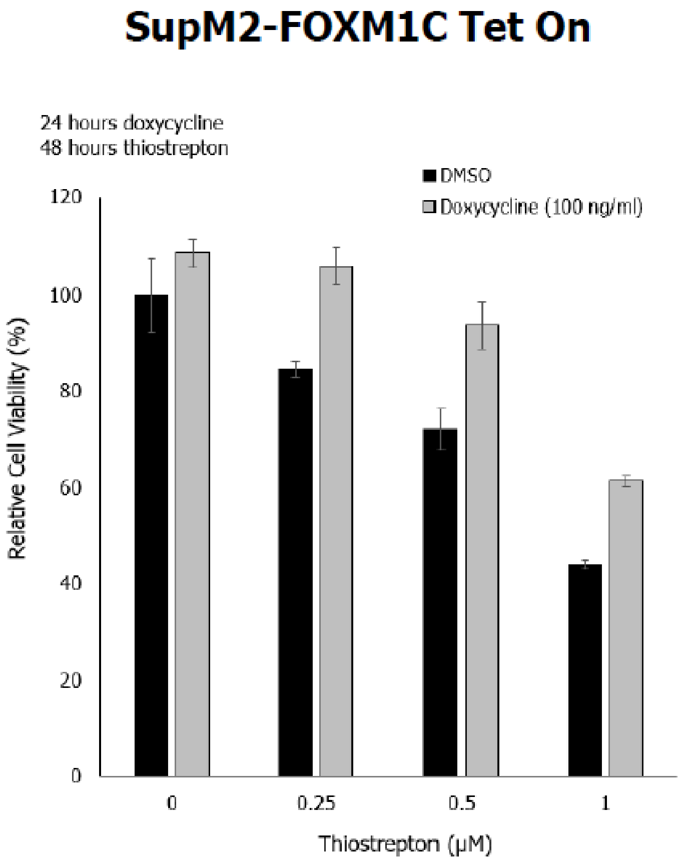

Figure S4. Overexpression of FOXM1 provides resistance to thiostrepton mediated growth inhibition of FOXM1 in a tetracycline inducible FOXM1 SupM2 cell line.

Figure 1A

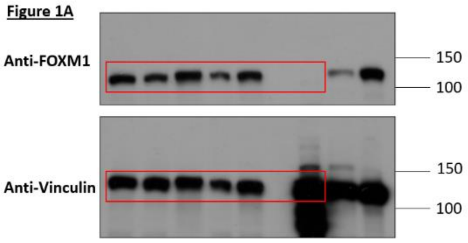

Figure 1B

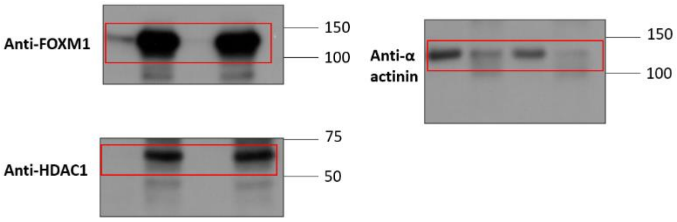

Figure 3B

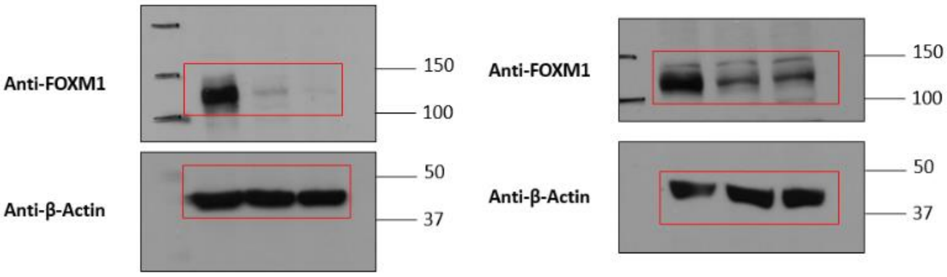

Figure 4B

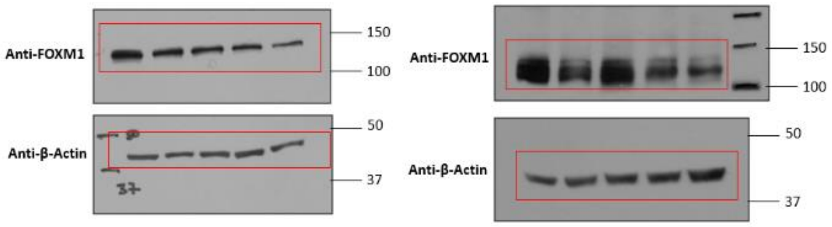

Figure 4C

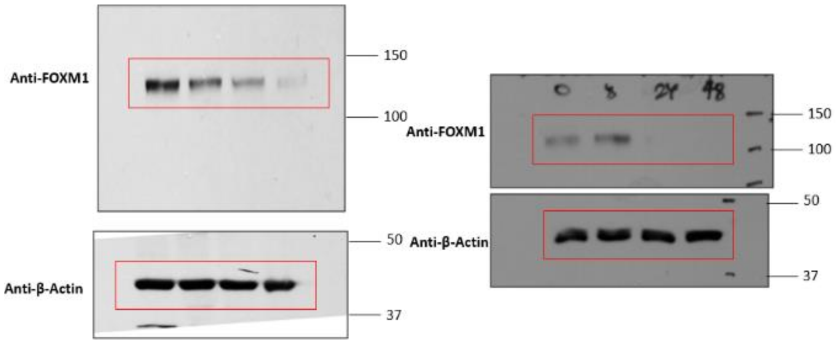

Figure 5A

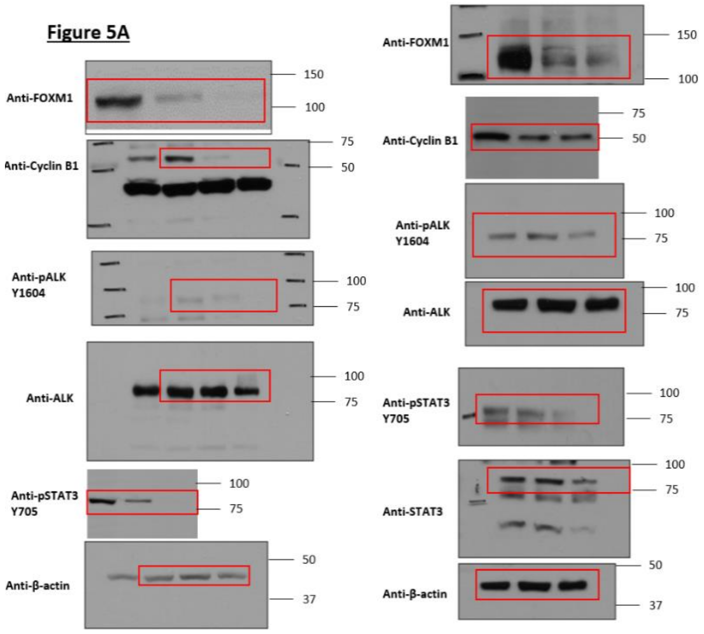

Figure 5B

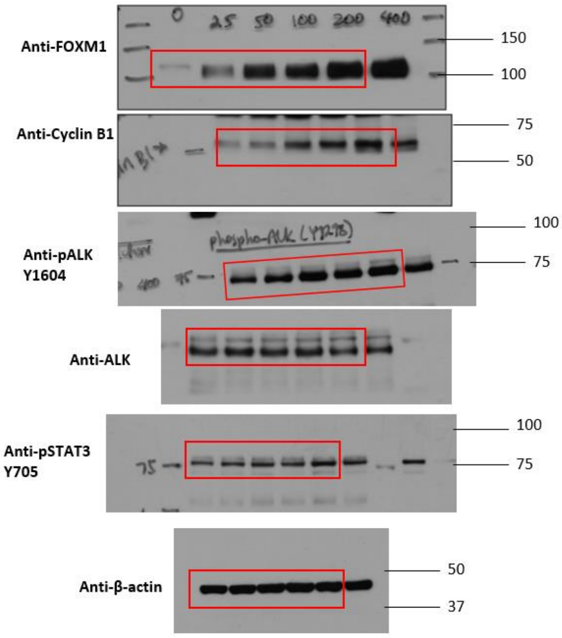

Figure 6

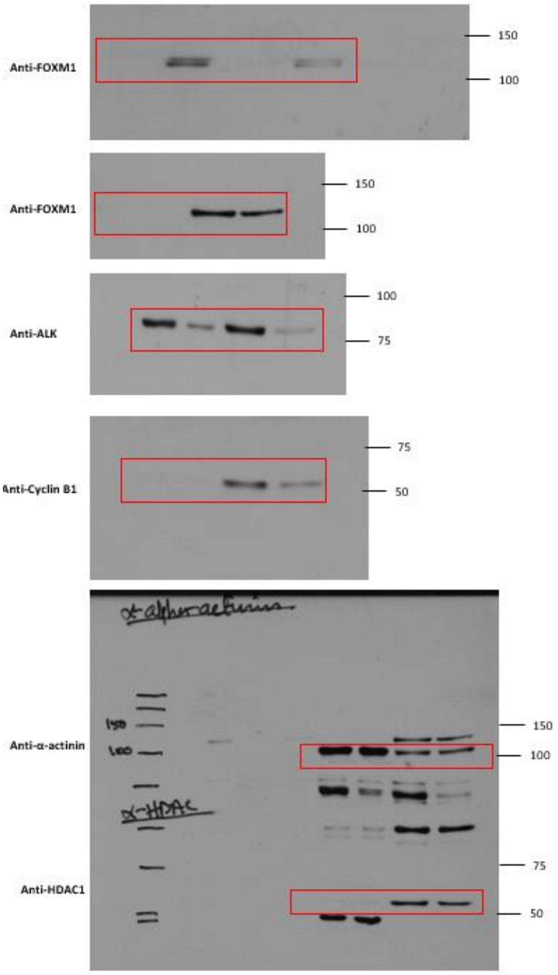

Anti-FOXM1
150
100
Anti-FOXM1
150
100
Anti-ALK
100
75
Anti-Cyclin B1
75
50
Anti-α-actinin
Anti-HDAC1
150
100
75
50

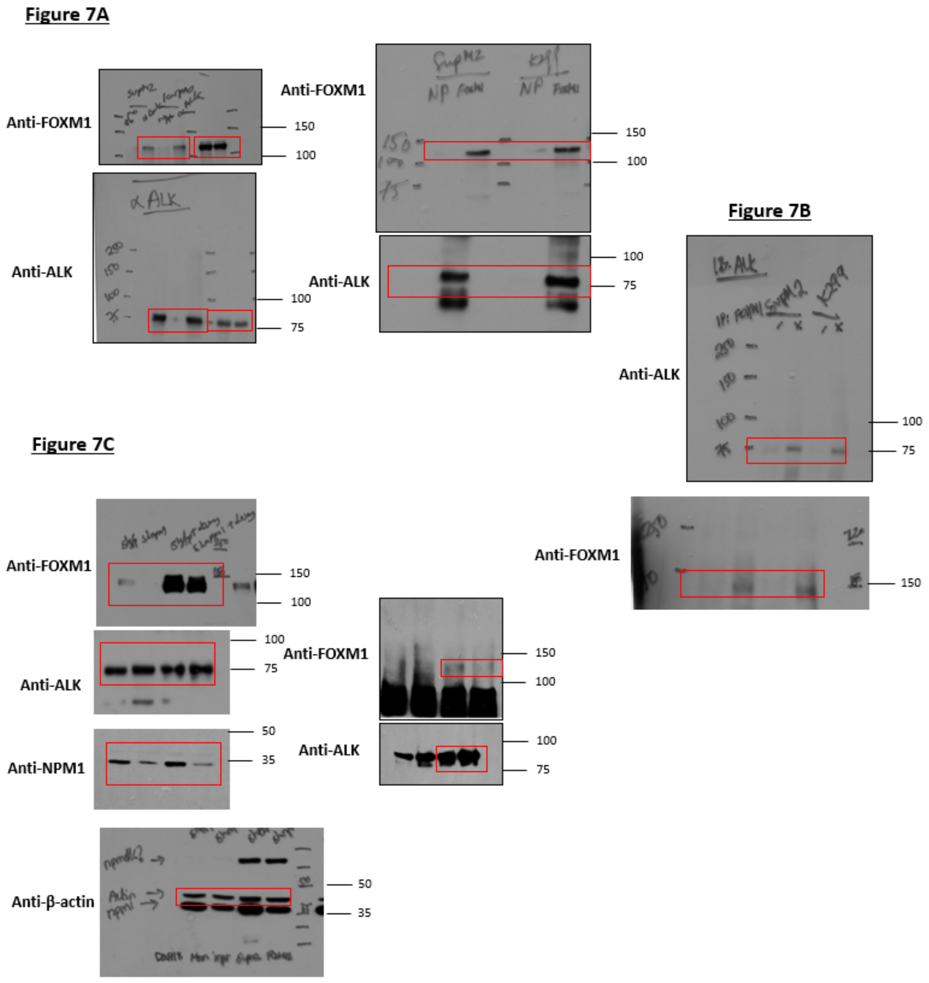

Figure S5. Uncropped western blot films.

Table S1. PCR primer sets used in the study.

**RT-PCR Primer Sequences (Figure 1B)**	
**Primers**	**Sequence**
FOXM1-isoforms (F)	CACCCCAGTGCCAACCGCTA
FOXM1-isoforms (R)	AAAGAGGAGCTATCCCCTCCTCAG
GAPDH (F)	GGTCTCCTCTGACTTCAACAGCG
GAPDH (R)	ACCACCCTGTTGCTGTAGCCAA
**Quantitative RT-PCR Primer Sequences (Figure 2A)**	
**Primers**	**Sequence**
FOXM1 (F)	CGTCGGCCACTGATTCTCAAA
FOXM1 (R)	GGCAGGGGATCTCTTAGGTTC
GAPDH (F)	GGTCTCCTCTGACTTCAACAGCG
GAPDH (R)	ACCACCCTGTTGCTGTAGCCAA
**ChIP PCR Primer Sequences (Figure 6C)**	
**Primers**	**Sequence**
Cyclin B1 Prom 1 (F)	CGCGATCGCCCTGGAAACGCA
Cyclin B1 Prom 1 (R)	CCCAGCAGAAACCAACAGCCGT
Cyclin B1 Prom 2 (F)	CCTCCAACCCAGAGAGTTGTTGC
Cyclin B1 Prom 2 (R)	AGCCAAGGACCTACACCCAGCA

© 2019 by the authors. Licensee MDPI, Basel, Switzerland. This article is an open access article distributed under the terms and conditions of the Creative Commons Attribution (CC BY) license (http://creativecommons.org/licenses/by/4.0/).
